# 1,25-Dihydroxyvitamin D3 Suppresses TLR8 Expression and TLR8-Mediated Inflammatory Responses in Monocytes In Vitro and Experimental Autoimmune Encephalomyelitis In Vivo

**DOI:** 10.1371/journal.pone.0058808

**Published:** 2013-03-14

**Authors:** Bo Li, David J. Baylink, Chandra Deb, Claudia Zannetti, Fatima Rajaallah, Weirong Xing, Michael H. Walter, K.-H. William Lau, Xuezhong Qin

**Affiliations:** 1 Department of Medicine, Loma Linda University School of Medicine, Loma Linda, California, United States of America; 2 International Agency for Research on Cancer, Lyon, France; 3 Musculoskeletal Disease Center, Jerry L. Pettis Memorial Veterans Affairs Medical Center, Loma Linda, California, United States of America; University of Lyon, France

## Abstract

1,25-Dihydroxyvitamin D3 (1,25(OH)_2_D_3_) suppresses autoimmunity and inflammation; however, the mechanism of its action has not been fully understood. We sought in this study to determine whether the anti-immune/anti-inflammatory action of 1,25(OH)_2_D_3_ is in part mediated through an interplay between 1,25(OH)_2_D_3_ and toll-like receptor (TLR)7/8 signaling. 1,25(OH)_2_D_3_ treatment prior to and/or following experimental autoimmune encephalomyelitis (EAE) induction effectively reduced inflammatory cytokine expression in the spinal cord and ameliorated EAE. These effects were accompanied with a reduction in expression of several TLRs with the most profound effect observed for TLR8. The expression of TLR8 adaptor protein MyD88 was also significantly reduced by 1,25(OH)_2_D_3_. To determine the molecular mechanism by which 1,25(OH)_2_D_3_ suppresses EAE induction of TLR8 and inflammatory cytokine expression, we evaluated whether 1,25(OH)_2_D_3_ can directly inhibit TLR8 signaling and the resulting inflammatory responses in human THP-1 monocytes. 1,25(OH)_2_D_3_ treatment not only significantly reduced TLR8 expression but also the expression or activity of MyD88, IRF-4, IRF-7 and NF-kB in monocytes challenged with TLR8 ligands. TLR8 promoter-luciferase reporter assays indicated that 1,25(OH)_2_D_3_ decreases TLR8 mRNA level in part via inhibiting TLR8 gene transcription activity. As a result of inhibition on TLR8 signaling cascade at various stages, 1,25(OH)_2_D_3_ significantly diminished the TLR8 target gene expression (TNF-α and IL-1β). In summary, our novel findings suggest that TLR8 is a new target of 1,25(OH)_2_D_3_ and may mediate the anti-inflammatory action of 1,25(OH)_2_D_3_. Our findings also point to a destructive role of TLR8 in EAE and shed lights on pathogenesis of multiple sclerosis.

## Introduction

Multiple sclerosis (MS) is a debilitating disease of the central nerve system (CNS) which affects more than 2.5 million people worldwide every year [1]. The pathological hallmarks of MS are demyelination, multifocal inflammation, axonal degeneration and oligodendrocyte loss [1], which have been suggested to be associated with infection [2]. Current therapies use mostly disease-modifying drugs, focusing on blocking and regulating systemic functions and CNS infiltration of immune cells [3], [4]. However, these therapies only attenuate or delay MS symptoms and are not effective in halting the disease progression [3], [4]. Thus, a thorough understanding of the pathogenesis of MS could lead to identification of new drug targets for treatment of MS.

Experimental autoimmune encephalomyelitis (EAE) is a primary T-cell-mediated disorder that has provided important insights into the pathogenesis and treatment of MS. Similar to what occurs in MS, excessive production of inflammatory cytokines by activated microglia and invading inflammatory cells appear to play a critical role [1], [5]. In this regard, treatment of EAE mice with 1,25-dihydroxyvitamin D3 (1,25(OH)_2_D_3_), the active form of vitamin D, was able to suppress inflammatory cytokine production in the inflamed EAE spinal cords and effectively ameliorate EAE [6], [7], [8], [9], [10], [11]. Identification of the molecules which mediate the anti-immune/inflammatory action of 1,25(OH)_2_D_3_ may uncover new mechanisms by which this hormone inhibits inflammatory cytokine production.

One pathway that leads to inflammation of CNS under an autoimmune condition could be the overly activation of toll-like receptors (TLRs), which leads to production of inflammatory cytokines via activating NF-kB and IFN regulatory factor (IRF) dependent pathway [12], [13], [14], [15]. Although several in vitro studies demonstrate that 1,25(OH)_2_D_3_ was able to reduce TLR2, 4 and 9 expression [16], [17], [18], [19], no information is available on the role of 1,25(OH)_2_D_3_ in modulating the action of other important TLRs in vitro or any of TLRs in vivo. TLR7 and 8 belong to the same subfamily and both are located in the intracellular endosomal compartments [20]. These TLRs recognize viral infection in the form of foreign nucleic acids [20]. TLRs can also be activated by endogenous ligands that are released from damaged cells [12], [21]. Since MS has been suggested to be associated with infection [2], TLR7 and 8, which are activated by viral pathogen penetrating the host cells, are of particular relevance to pathogenesis of EAE. Currently information on whether these TLRs, especially the TLR8, play a protective or detrimental role in EAE is scarce. TLR8 is known to be expressed in microglia and neurons [22], [23] and activation of TLR8 by agonists in cultured cortical neurons inhibited neurite outgrowth and induced apoptosis [22]. In vivo, intracerebroventricular inoculation of newborn mice with the TLR7/8 agonist (R837) induced neuroinflammation and production of proinflammatory cytokines and chemokines [24]. In addition, expression of both TLR7 and TLR8 in the spinal cord was increased upon onset of the EAE [25]. In contrast to these studies, which in general suggest a destructive role of TLR8 in CNS, systemic TLR8 gene deletion in mice led to development of autoimmunity and glomerulonephritis [26].This discrepancy further sparked our interest to investigate the role and regulation of this intriguing TLR under an autoimmune condition.

The primary objective of this study was to evaluate if 1,25(OH)_2_D_3_ acts to suppress the inflammation in part by regulating the TLR8 signaling. We found that TLR8 was increased by EAE to the largest extent among the several TLRs analyzed. Importantly this up-regulation was abolished by treatment of EAE mice with 1,25(OH)_2_D_3,_ which ameliorated EAE and reduced expression of key inflammatory cytokines. Our in vitro results are in line with these in vivo data and demonstrate that 1,25(OH)_2_D_3_ significantly inhibited TLR8 signaling at multiple stages thereby counteracting the TLR8-induced TNF-α and IL-1β expression. These novel findings suggest that TLR8 is a new target of 1,25(OH)_2_D_3_ and may mediate the anti-inflammatory action of 1,25(OH)_2_D_3_ in EAE.

## Materials and Methods

### Reagents

Myelin oligodendrocyte glycoprotein (MOG)_35–55_ peptide was purchased from CS Biotechnology Company (Menlo Park, CA). Cell culture reagents used were: RPMI 1640, glutamine, penicillin-streptomycin, obtained from Invitrogen (Carlsbad, CA); FBS from Hyclone (Logan, UT); 2-mercaptoethanol from Sigma-Aldrich (St. Louis, MO). The TLR8 ligand CL075 and ssRNA (ssRNA40/Lyovec) were purchased from Invivogen (San Diego, CA). 1,25(OH)_2_D_3_ and peanut oil were purchased from Sigma-Aldrich (St. Louis, MO). The antibodies used for immunohistochemistry were: anti-F4/80 from AbD Serotec (Raleigh, NC); anti-TLR8 from Abcam (Cambridge, MA). The antibodies used for FACS were: PE-conjugated anti-TLR8, PE-conjugated anti-TLR7 and PE-conjugated rabbit IgG isotype control from Imgenex (San Diego, CA); an PE-conjugated mouse IgG1 isotype control from BD Pharmingen (San Jose, CA). The antibodies used for western blotting were: anti-NF-kB, anti-IkB, and anti-β-actin from Abcam (Cambridge, MA).

### Mice

Female C57BL/6 mice were purchased from The Jackson Laboratory (Bar Harbor, ME). All mice were used at ages 8–12 weeks. The investigators adhered to the Animal Welfare Act Regulations and other Federal statutes relating to animals and experiments involving animals and the principles set forth in the current version of the Guide for Care and Use of Laboratory Animals, National Research Council. All experiments were performed according to protocols approved by the Institutional Animal Care and Use Committee at the Loma Linda University.

### 1,25(OH)_2_D_3_ Treatment and EAE Induction

For the prevention studies, mice were injected i.p. with 2 µg 1,25(OH)_2_D_3_ in peanut oil 24 hours prior to injection of MOG. From day 3 post-MOG injection, mice were injected i.p. with a reduced dose of 1,25(OH)_2_D_3_ (100 ng/mouse) every other day till the end of the experiment. For the treatment studies, 200 ng of 1,25(OH)_2_D_3_ in peanut oil was injected i.p for every 3 days from day 7 post-MOG injection. In both studies, peanut oil only served as the placebo vehicle control. MOG-immunized mice without 1,25(OH)_2_D_3_ administration developed EAE around 10 days post-MOG injection, and disease usually peaked around 3 weeks post-MOG immunization. In our experiments, all the mice were sacrificed at day 14 post-MOG injection. To induce EAE, mice were immunized subcutaneously with 200 µg MOG emulsified in complete Freund’s adjuvant (Difco, Lawrence, KS) supplemented with 5 mg/ml Mycobacterium tuberculosis H37Ra (Difco, Lawrence, KS). The mice were injected twice i.p. with 200 ng of pertussis toxin (List Biological Laboratories, Campbell, CA) at the time of immunization and 48 hours later. The mice were then monitored daily for clinical symptoms of disease, and disease severity was scored on a numerical scale from 0–5 as follows: 0, no disease; 1, weak tail or wobbly walk; 2, hind limb paresis; 3, hind limb paralysis; 4, hind and forelimb paralysis; 5, death. Paralyzed mice were given easy access to food and water.

### Cell Culture

Human monocytic THP-1 cells, obtained from American Type Culture Collection (Manassas, VA) were cultured in RPMI-1640 medium supplemented with 10% fetal bovine serum (FBS), 2 mM glutamine, 0.05 mM 2-mercaptoethanol and 100 µg/ml penicillin and streptomycin in 24-well plates in a humidified atmosphere containing 5% CO_2_ at 37°C. For the THP-1 cells treatment, the cells were seeded at a density of 1×10^6^ cells/ml and concentration of 1,25(OH)_2_D_3_ routinely used was 100 nM. Thirty min after 1,25(OH)_2_D_3_ addition, CL075 or ssRNA was added to the final concentration (1 µg/ml for CL075 and 5 µg/ml for ssRNA). After additional 20 h incubation, cells were harvested for total RNA isolation or FACS analysis.

### Quantitative Real-time RT-PCR

mRNA levels of the genes of interest in the spinal cord and THP-1 cells were analyzed by real-time RT-PCR. Total RNA was isolated using TRIzol reagent (Invitrogen, Carlsbad, CA) and purified with RNeasy mini kit (Qiagen, Valencia, CA). cDNA was prepared using the superscript III cDNA synthesis kit (Invitrogen, Carlsbad, CA), according to instructions. Specific primers were synthesized by Integrated DNA Technologies (San Diego, CA) as shown in **Table S1**. Real-time PCR was performed with SYBR Green PCR Master Mix (Applied Biosystems, Carlsbad, CA) in 7500 Fast Real Time PCR System (Applied Biosystems, Carlsbad, CA). Each reaction was run in triplicate. The data were normalized to GAPDH or β-actin as a reference and presented as fold change relative to control samples.

### Immunohistochemistry

Mice were euthanized and perfused with PBS. The spinal cords were removed and fixed by immersion in 4% paraformaldehyde (PFA) overnight at 4°C, followed by cryopreservation in 30% sucrose in PBS at 4°C until the tissues got submerged. The spinal cords were embedded in optimal cutting temperature (OCT) compound (Sakura Finetek, Torrance, CA) and frozen under liquid nitrogen. Sections (8 µm) were cut on a cryostat, thawed-mounted on Superfrost plus slides, air dried, and stored at –20°C for further use. After fixation with 4% PFA and endogenous peroxidase activity blockage, tissue sections were blocked with 10% normal goat serum (Sigma-Aldrich, St. Louis, MO) for 1 h at RT and then incubated with primary antibodies that were diluted (anti-TLR8 monoclonal antibody 1:150 and anti-F4/80 polyclonal antibody 1:200) in PBS containing 2% normal goat serum overnight at 4°C. Samples were subsequently incubated with biotinylated species-specific secondary antibodies (1:300 dilution, Vector Laboratories, Burlingame, CA) for 30 min at RT followed by incubation with HRP (1:300 dilution, Vector Laboratories, Burlingame, CA) for 30 min at RT. Staining was visualized using 3,3′-diaminobenzidine (DAB) substrate (Vector Laboratories, Burlingame, CA) and counterstained with haematoxylin (Sigma-Aldrich, St. Louis, MO).

### Flow Cytometry

The abundance of TLR8 and TLR7-positive cells in spinal cord was determined using a FACSAria flow cytometer (BD Biosciences, San Jose, CA). Mice were perfused intracardially with PBS to remove blood cells from the organs. Spinal cords were collected and dissociated to single-cell suspension using neural tissue dissociation kits (Miltenyi Biotec, Auburn, CA). Mononuclear cells in the spinal cord digests were isolated by 30%/70% Percoll gradient centrifugation, washed with PBS, fixed in 2% PFA for 1 h at 4°C and then permeablized in a buffer containing 0.1% saponin. Nonspecific antibody binding to Fc receptors was blocked by incubation in 0.1% saponin containing 2% goat serum for 30 min at room temperature. Cells were then incubated with PE-conjugated anti-TLR8 antibody, PE-conjugated anti-TLR7 antibody or isotype control for 3 hours at room temperature in dark. Cells were washed twice with permeabilization buffer and once with PBS prior to FACS analysis. Isotype primary conjugated antibodies served as a negative control. Data were analyzed using FACSDiva software (BD Biosciences, San Jose, CA).

### ELISA

Serum concentration of IL-17 was measured by an ELISA kit (R&D Systems, Minneapolis, MN) according to the manufacturer’s protocol.

### Western Immunoblot Analysis

THP-1 cells were collected after treatment with an indicated dose of CL075 and/or 1,25(OH)_2_D_3_, washed twice with ice-cold PBS, and lysed on ice for 20 min in lysis buffer containing 25 mM Tris^.^HCl (pH 7.6), 150 mM NaCl, 1% NP-40, 1% sodium deoxycholate, 0.1% SDS, phosphatase inhibitors cocktail 2 and protease inhibitor cocktail (Sigma-Aldrich, St. Louis, MO). Supernatants were collected after centrifugation at 13,000 rpm for 20 min at 4°C. Protein concentration in the supernatants was measured using a Bio-Rad protein assay kit. Equal amounts of cellular protein were resolved on a 10% SDS-PAGE gel and then transferred to nitrocellulose membrane by electrophoresis. After blocking with 5% dry milk in PBS/Tween 20 (0.1%) for 16 hr at 4°C, membranes were incubated with antibodies specific to NF-kB and IkB followed by horseradish peroxidase-conjugated secondary antibodies incubation for 1 h. After washing with PBS/Tween 20, signals were visualized with autoradiography using ECL system. To control for protein loading, the membranes were reprobed with anti-β-actin antibody.

### Transfection and Luciferase Reporter Assay

The cloning of the 3.8 kb 5′ regulatory sequence (promoter) of the human TLR8 gene and construction of the TLR8 promoter-luciferase report plasmid were described in a previous study [27]. The investigators adhered to the current version of the National Institutes of Health (NIH) Guidelines for Research Involving Recombinant DNA Molecules.

THP-1 cells were co-transfected with indicated luciferase reporter plasmids and TK-Renilla plasmid at ratio of 10:1 using X-tremeGENE HP DNA transfection reagent (Roche Applied Science, Indianapolis, IN). After 24 h transfection, cells were stimulated for 20 h with 4 µg/ml CL075 in the absence or presence of 100 nM 1,25(OH)_2_D_3_. The cells were washed once with PBS, lysed, and luciferase activity was measured using the Dual-Luciferase Reporter Assay System (Promega, Madison, WI).

### Statistical Analysis

Data were reported as mean ± standard error of the mean (SEM). The statistical significance of differences between the group means was assessed using the Student’s t test or the Mann-Whitney U test. *P*<0.05 was considered as statistically significant.

## Results

### 1,25(OH)_2_D_3_ Treatment Reduced Inflammatory Cell Infiltration and Inflammatory Cytokine Expression in the EAE Spinal Cord

**Figure 1:**
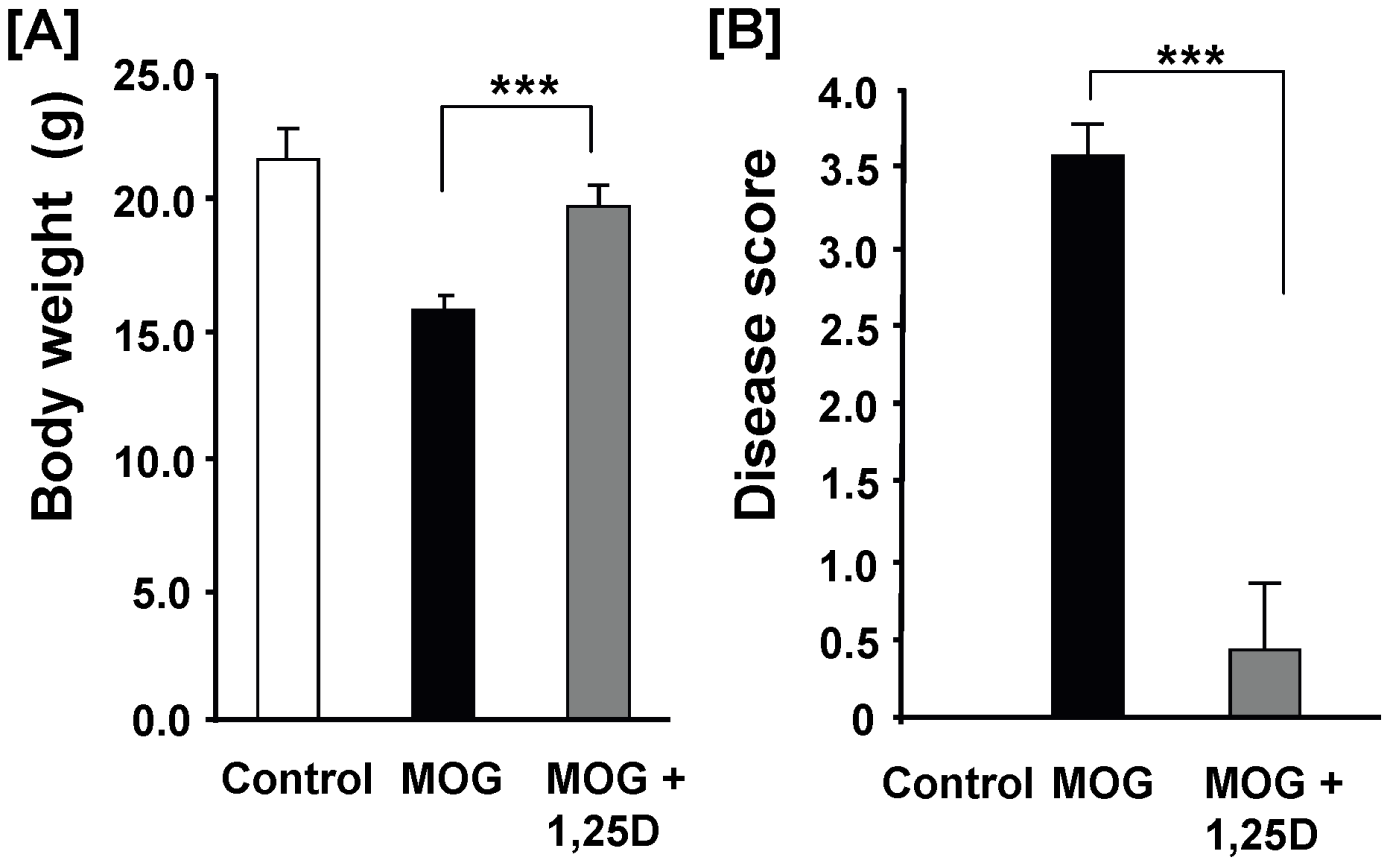
1,25(OH)_2_D_3_ treatment significantly protected mice from development of EAE. EAE was induced by immunonization with MOG (*Material & Methods*). Mice were injected i.p. with 2 µg of 1,25(OH)_2_D_3_ one day before MOG injection and then 0.1 µg 1,25(OH)_2_D_3_ every other day till day 14 post-MOG injection. Body weight (**A**) and clinical score (**B**) were determined at day 14 post-MOG injection. Data represent the means ± SEM (n = 7) from two independent experiments. *** *P*<0.001.

In order to evaluate the effect of 1,25(OH)_2_D_3_ on TLR8 signaling and its potential role in the pathogenesis of EAE, it is important to evaluate if 1,25(OH)_2_D_3_ treatment under our experimental conditions is effective in ameliorating EAE. In the prevention model, mice received a single injection of 1,25(OH)_2_D_3_ one day prior to and multiple injections of 1,25(OH)_2_D_3_ post-MOG injection (Material and Methods). 1,25(OH)_2_D_3_ treatment reversed the decrease in body weight of EAE mice (**Figure 1A**). At day 14, there was no significant difference in body weight of control mice vs. EAE mice treated with 1,25(OH)_2_D_3._ All of the 7 MOG-injected mice developed severe EAE symptoms with an average disease score of 3.5 (**Figure 1B**). In contrast, only one of the 7 MOG-injected mice treated 1,25(OH)_2_D_3_ developed a moderate EAE symptom (**Figure 1B**). The clinical score of an independent experiment in prevention model was recorded daily and presented in the **Figure S1A**.

**Figure 2:**
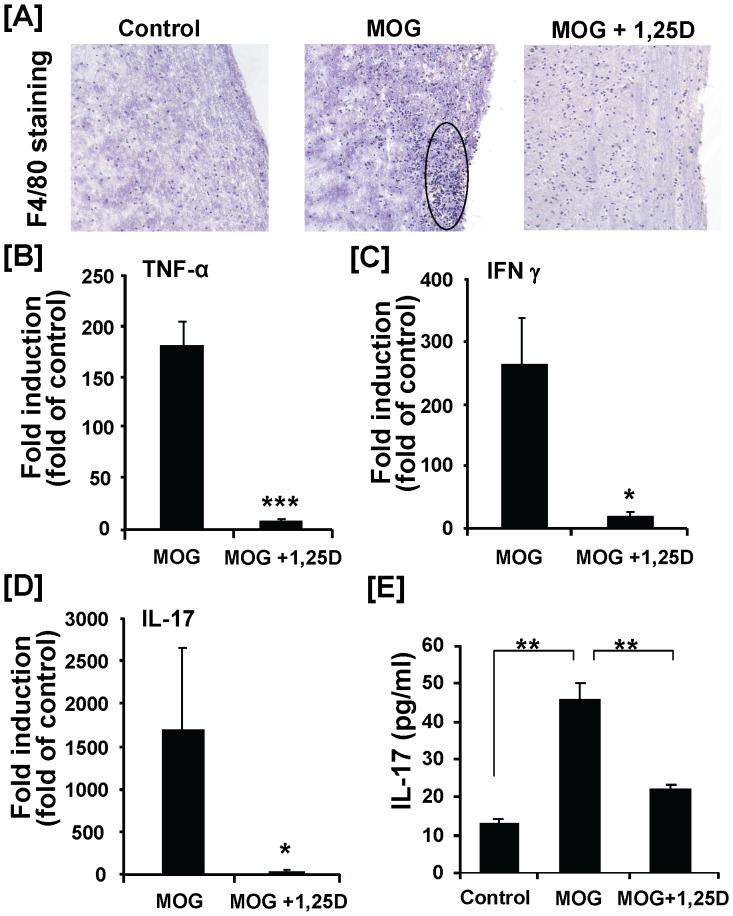
Suppressive effect of 1,25(OH)_2_D_3_ on inflammatory cell infiltration and inflammatory cytokine production in MOG-induced EAE. Animal treatments were described in Figure 1 legend. Fourteen days after MOG injection, spinal cords were collected for RNA isolation and immunohistochemical analysis. (**A**) Representative F4/80 immunostaining of frozen spinal cord sections. Spinal cords from MOG treated mice showed dense mononuclear cell infiltration (circle). The number of F4/80-positive cells were reduced following treatment of EAE mice with 1,25(OH)_2_D_3_. Three mice were analyzed in each group. Original magnification was X10. (**B–D**) Real-time RT-PCR analysis of the inflammatory gene expression (TNF-α, INF-γ and IL-17) in spinal cord. (**E**) Serum concentration of IL-17 was determined by ELISA. Data represent the means ± SEM (n = 4) from one of two independent experiments. * *P*<0.05; ** *P*<0.01; *** *P*<0.001.

Immunostaining of the spinal cord sections for microglia/macrophage maker F4/80 revealed a severe inflammatory cell infiltration in the EAE mice compared with the control mice (**Figure 2A**). The inflammatory cell infiltration, mainly in the white matter, was essentially prevented by 1,25(OH)_2_D_3_ (**Figure 2A**). mRNA expression of three key inflammatory cytokines (TNF-α, INF-γ and IL-17) in spinal cord was significantly increased in mice immunized with MOG (**Figure 2B–D**). In addition, EAE mice showed a significant increase in serum concentration of IL 17, the most important disease-driving pro-inflammatory cytokine in this model (**Figure 2E**). All of these changes were significantly reduced by 1,25(OH)_2_D_3_ treatment (**Figure 2B–E** **)**.

In the treatment model, mice were injected, i.p. with 1,25(OH)_2_D_3_ for 7 days beginning from day 7 post-MOG injection. We chose to initiate 1,25(OH)_2_D_3_ treatment at day 7 as it has been reported that inflammatory cell infiltration and inflammatory cytokine expression in the spinal cord were increased at this disease stage [25], [28]. Moreover, our experiment indicated that expression of TLR8 was significantly increased as early as 5 days post-MOG injection (data not shown). The experiment was terminated at day 14 because most of the MOG-immunized mice developed severe disease symptoms at this time evidenced by paralysis of both hind limbs, whereas the disease progression was essentially halted by the 7-day 1,25(OH)_2_D_3_ treatment (**Figure S1B and S2A**).

1,25(OH)_2_D_3_ treatment for 7 days beginning at day 7 post-MOG immunization significantly attenuated the EAE-induced increase in IL-17 mRNA level in the spinal cord (**Figure S2B**) as well as IL17 concentration in the serum (**Figure S2C**). Subsequently, modulation of TLR expression by EAE and 1,25(OH)_2_D_3_ treatment were evaluated in both models (see section below).

### TLR8 Expression in the Spinal Cord was Markedly Increased by EAE and this Increase was Significantly Reduced by 1,25(OH)_2_D_3_ Treatment

**Figure 3:**
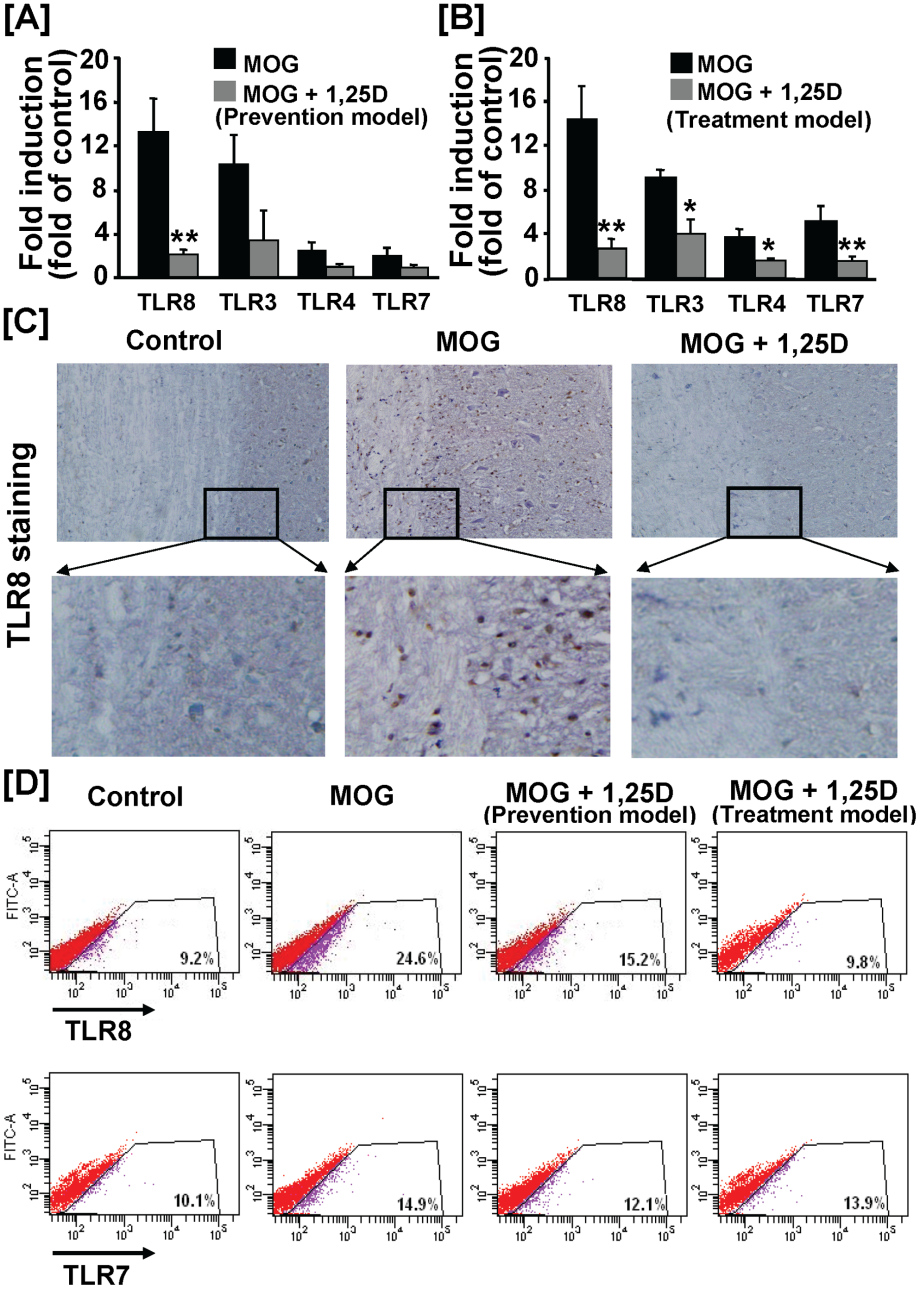
Treatment with 1,25(OH)_2_D_3_ reduced EAE-induced expression of TLR8 in the spinal cord. (**A**) Real-time RT-PCR analysis of expression of the key TLRs in the spinal cord (Prevention model, refer to Figure 1 legend for 1,25(OH)_2_D_3_ treatment scheme). The mRNA levels of TLR3 and TLR8 were significantly increased in EAE spinal cords. 1,25(OH)_2_D_3_ only significantly reduced TLR8 mRNA level. Data shown are the means ± SEM (n = 4) from one of two independent experiments. (**B**) Real-time RT-PCR analysis of expression of the key TLRs in the spinal cord (Treatment model). Mice were injected with 1,25(OH)_2_D_3_ beginning at day 7 post-MOG injection (*Material and Methods*). Expression of all 4 TLRs analyzed were significantly induced by EAE and reduced by 1,25(OH)_2_D_3_ treatment. Data shown are the means ± SEM (n = 4) from one of two independent experiments. In both treatment and prevention models, TLR8 mRNA level showed the highest magnitude of induction by EAE and was significantly down-regulated by 1,25(OH)_2_D_3_ treatment. * *P*<0.05; ** *P*<0.01. (**C**) Representative TLR8 immunostaining of spinal cord sections. Animal treatments were described in Figure 1 legend. Three mice were analyzed in each group. The magnification was X10 (upper panels). (**D**) FACS analysis of intracellular TLR7 and 8 expressions in the spinal cord in both models. Mononuclear cells were isolated using pooled spinal cords from 2–3 mice each group by Percoll gradients (*Material and Methods*). Data are representative of two independent analyses.

In the prevention model, among the 4 TLRs analyzed, TLR3 and TLR8 showed a significant increase in their steady state mRNA levels in the EAE spinal cords compared to the control spinal cords (10–12 fold) (**Figure 3A**). Treatment of EAE mice with 1,25(OH)_2_D_3_ only significantly decreased TLR8 mRNA level (**Figure 3A**). In the treatment model, the mRNA levels of TLR3, 4, 7 and 8 were all significantly increased by EAE. 1,25(OH)_2_D_3_ treatment significantly decreased the EAE-induced expression of all 4 TLRs analyzed (**Figure 3B**). Similar to results obtained in the prevention model, TLR8 showed the highest magnitude of induction by EAE and reversal by 1,25(OH)_2_D_3_ treatment (**Figure 3B**). For this reason, our subsequent analysis was focused on TLR8.

Consistent with real-time PCR analysis of the TLR8 mRNA, we found that the abundance of the spinal cord cells expressing TLR8 protein was significantly increased in the EAE spinal cord and this increase was attenuated by 1,25(OH)_2_D_3_ treatment, as determined by immunostaining of the spinal cord sections with TLR8 antibody (**Figure 3C**). To further confirm these results, the number of TLR8-positive mononuclear cells in the spinal cord was analyzed by FACS in both prevention and treatment models. The frequency of TLR8 positive cells in the spinal cord of EAE mice was increased by 2.7 fold compared to that of the control spinal cord (**Figure 3D**). This increase was reduced moderately in the prevention model and essentially blocked in the treatment model by 1,25(OH)_2_D_3_. In contrast, only a mild increase in abundance of TLR7-postive cells was observed in EAE spinal cord under our experimental conditions (**Figure 3D**). Collectively, these data demonstrate that 1,25(OH)_2_D_3_ not only reduced EAE-induced TLR8 expression but also the abundance of the spinal cord cells expressing TLR8.

**Figure 4:**
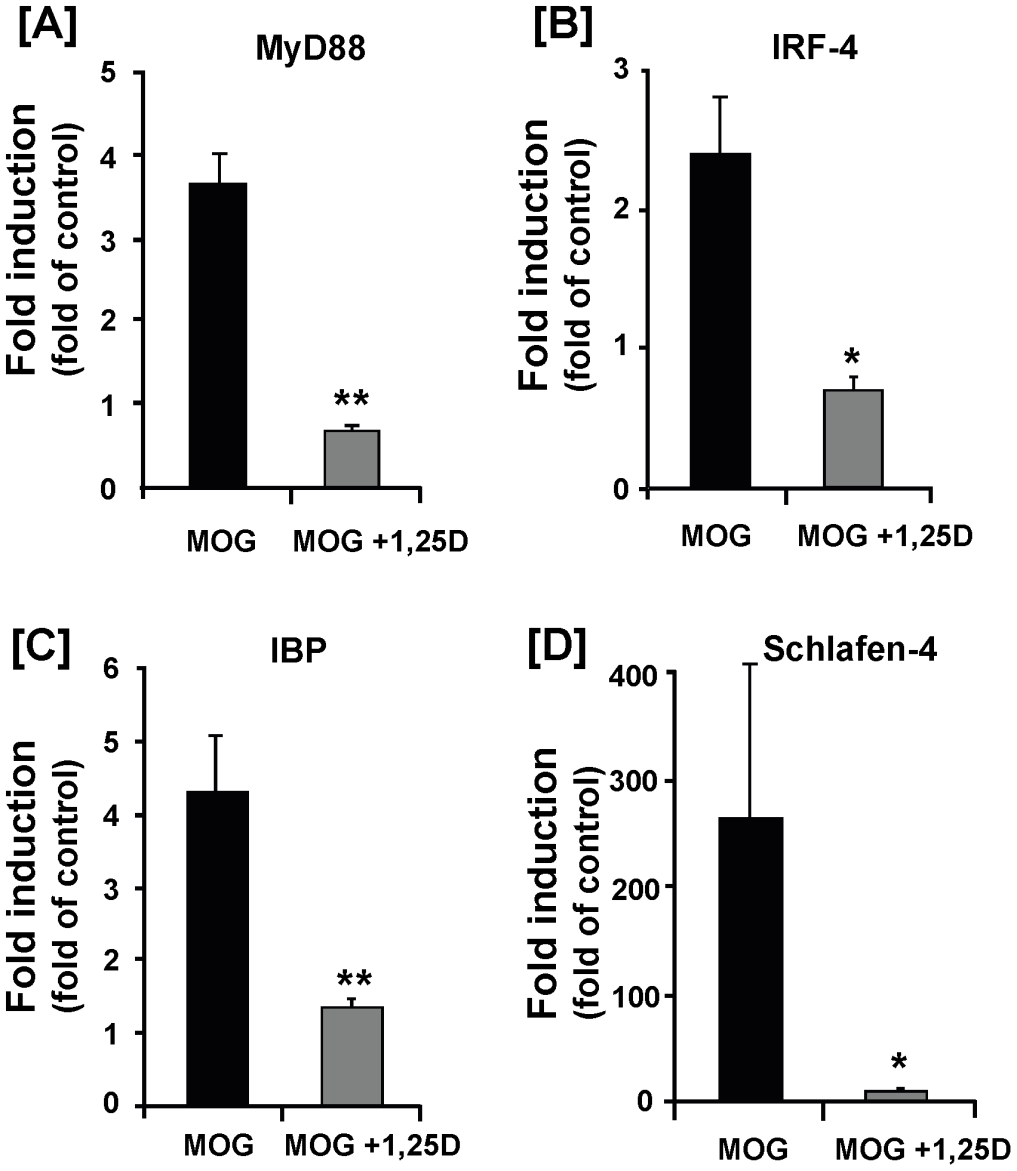
1,25(OH)_2_D_3_ inhibits activation of TLR8 signaling cascade in EAE. Animal treatments and spinal cord sample collections were described in Figure 1 legend. (**A–D**) Real-time RT-PCR analysis of the relative levels of mRNA for MyD88 (**A**), IRF-4 (**B**), IBP (**C**), and Schalfen-4 (**D**). These downstream mediators of TLR8 signaling showed significant increase of mRNA expression in EAE and this increase was completely prevented by 1,25(OH)_2_D_3_ treatment. Data represent the means± SEM (n = 4) from one of two independent experiments. * *P*<0.05; ** *P*<0.01.

Since MyD88 is an immediate downstream adaptor molecule that interacts with TLRs and its deficiency led to resistance to EAE development [29], [30], we evaluated whether its expression was also affected under EAE. In the prevention model, EAE increased MyD88 mRNA level by ∼4 fold and this increase was completely prevented by 1,25(OH)_2_D_3_ treatment (**Figure 4A**). A similar pattern of regulation was observed for other downstream mediators of TLR8 signaling, including IFN regulatory factor-4-binding protein (IBP), IRF-4, and Schlafen-4 (**Figure 4B–D**). In the treatment model, 1,25(OH)_2_D_3_ suppressed the EAE-induced expression of MyD88 and IRF-7 to the level measured in the control spinal cord (**Figure S2D–E**).

### 1,25(OH)_2_D_3_ Inhibited Expression of Inflammatory Cytokines and TLR8 in THP-1 Monocytes Challenged with TLR8 Ligands

**Figure 5:**
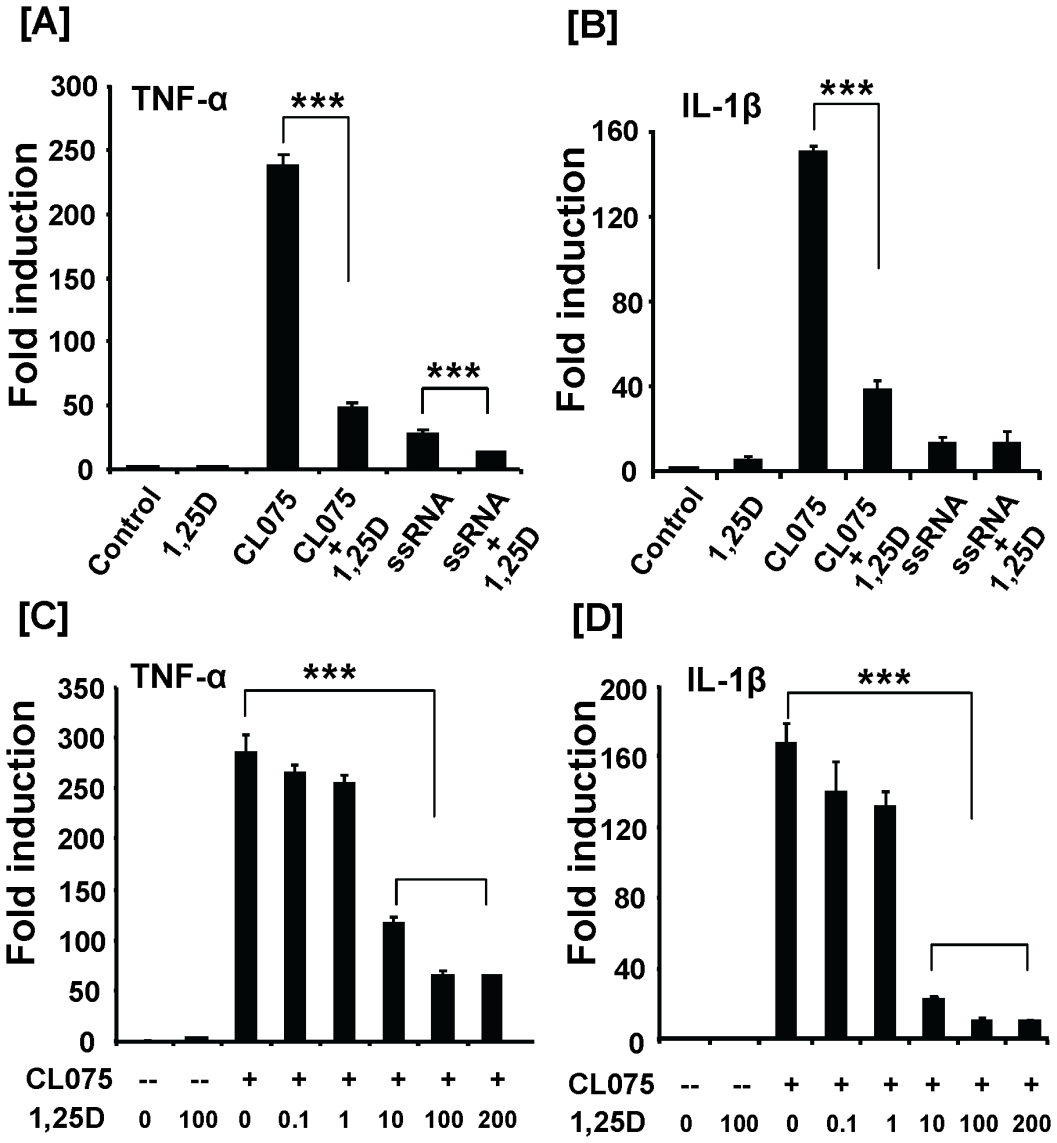
1,25(OH)_2_D_3_ inhibits TLR8-mediated inflammatory cytokine expression in human THP-1 monocytes. (**A–B**) Human monocyte THP-1 cells were pretreated with 1,25(OH)_2_D_3_ (100 nM) for 30 min and then stimulated with CL075 (1 µg/ml) or ssRNA (5 µg/ml) for 20 h. (**C–D**) THP-1 cells were pretreated with different doses of 1,25(OH)_2_D_3_ (0.1–200 nM) for 30 min and then incubated with CL075 (1 µg/ml) for additional 20 h. TNF-α and IL-1β mRNA levels were measured by real-time RT-PCR. Data shown are means ± SEM (n = 4) and are representative of three independent experiments. *** *P*<0.001.

To determine the mechanism by which 1,25(OH)_2_D_3_ suppresses EAE induction of TLR8 and inflammatory cytokine expression, we tested the possibility that 1,25(OH)_2_D_3_ may have exerted these effects by directly acting on inflammatory cells. Human monocytes (THP-1) were used in our studies because the TLR8 ligands/agonists are better defined in human than mouse species. Moreover, we possess human TLR8 promoter clone for evaluation of TLR8 transcriptional regulation in human cells [27]. Monocytes were challenged with TLR8 agonist/ligand to mimic the in vivo disease state in which self ligands generated from apoptotic/necrotic cells is likely the primary reason for EAE-induced TLR8 activation. Co-treatment with 1,25(OH)_2_D_3_ at 100 nM significantly reduced the mRNA level of both TNF-α and IL-1β that were induced by synthetic TLR8 agonist (CL075) (**Figure 5A–B**). The expression level of TNF-α but not IL-1β was also significantly inhibited by the natural TLR8 agonist (ssRNA) (**Figure 5A–B**). The CL075-induced TNF-α and IL-1β expression was inhibited in a dose-dependent manner by 1,25(OH)_2_D_3_ (**Figure 5C–D**).

**Figure 6:**
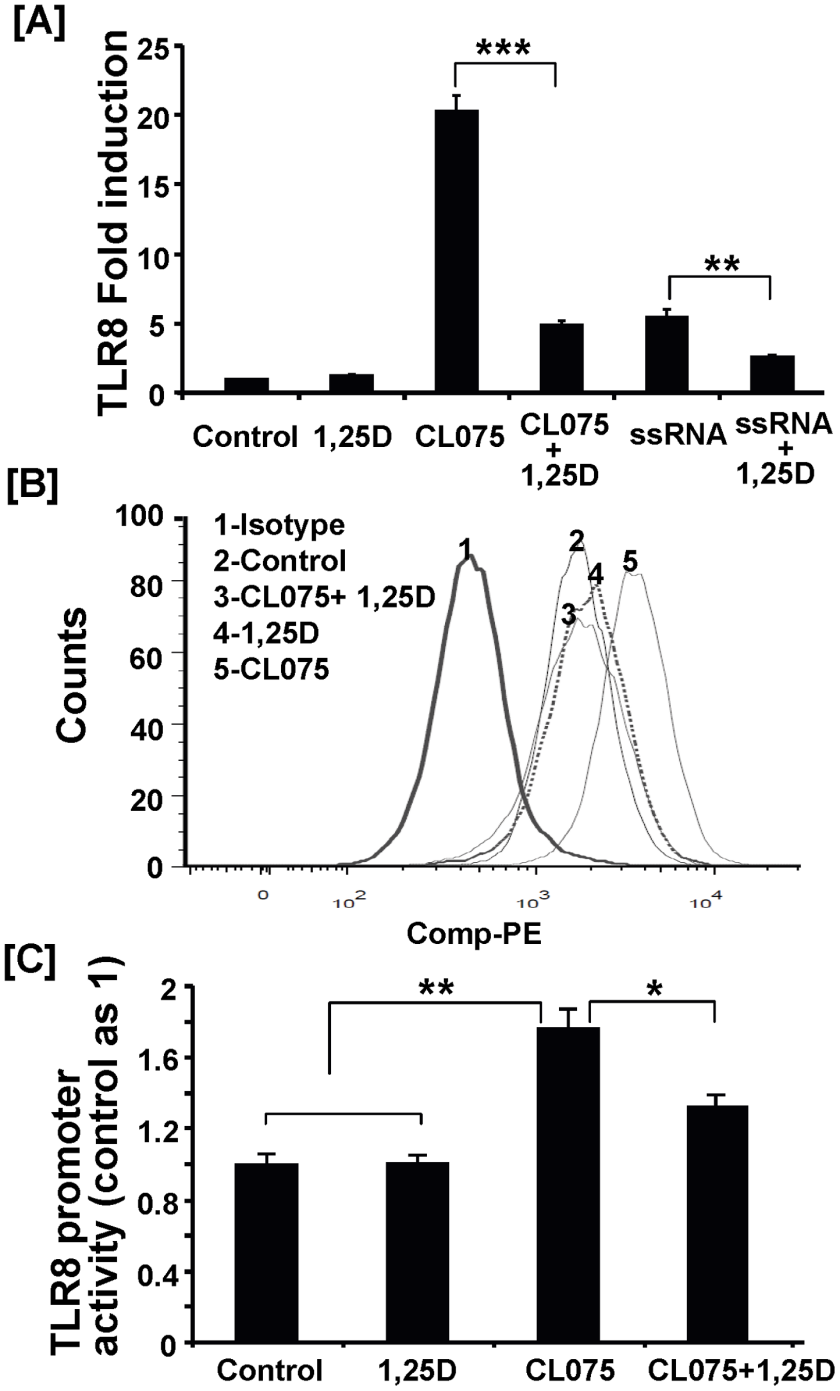
1,25(OH)_2_D_3_ inhibits TLR8 agonist-induced TLR8 expression in human monocytes. THP-1 cell treatment was described in Figure 5A-B legend. (**A**) mRNA level of TLR8 was subsequently determined by real-time RT-PCR. Data shown here represent means ± SEM (n = 4) and are representative of three independent experiments. (**B**) Intracellular expression of TLR8 was analyzed by FACS analysis using PE-conjugated anti-TLR8 antibody (*Material & Methods*). Isotype control antibody was used as negative control for non-specific TLR8 staining. A representative histogram plot was shown here from three independent experiments. (**C**) The THP-1 cells were co-transfected with TLR8 promoter-luciferase and TK-Renilla plasmid as described in *Material and Methods*. After 24 h, cells were pretreated with 1,25(OH)_2_D_3_ (100 nM) for 30 min and then stimulated with CL075 (4 µg/ml) for 20 h. The TLR8 promoter activity was presented as the ratio of luciferase activity to TK-Renilla activity. The data shown are means ± SEM (n = 4) and representative of two independent experiments. * *P*<0.05; ** *P*<0.01; ***, *P*<0.001.

In line with measurements of inflammatory cytokine expression, the steady state mRNA level of TLR8 was dramatically up-regulated by stimulation with synthetic or natural human TLR8 agonist, with a much stronger effect observed with the synthetic agonist CL075 (**Figure 6A**). The ligand/agonist-induced increase in TLR8 mRNA level was significantly reduced by 1,25(OH)_2_D_3_ treatment (**Figure 6A**). It should be noted that CL075 had no effect on TLR4 expression and had a much weaker effect on expression of TLR7 compared to that of TLR8 (**Figure S3**), suggesting that CL075 is relatively specific in up-regulating TLR8 expression.

To further confirm the inhibitory effect of 1,25(OH)_2_D_3_ on TLR8 expression, we analyzed the changes in the amount of intracellular TLR8 in monocytes by FACS analysis. Consistent with a lack of an effect of 1,25(OH)_2_D_3_ on basal TLR8 mRNA level, the basal level of TLR8 protein was not altered by 1,25(OH)_2_D_3_ treatment (**Figure 6B** **,** peak 2 vs. peak 4). CL075 stimulation increased the TLR8 signal strength (**Figure 6B**, peak 5 vs. peak 2). Following 1,25(OH)_2_D_3_ treatment, the agonist-induced shift in TLR8 signal returned to its basal level (**Figure 6B**, peak 5 vs. peak 3).

Next we determined whether 1,25(OH)_2_D_3_ decreases steady-state TLR8 mRNA level in part through inhibiting the TLR8 transcription activity using the recently cloned 5′ regulatory sequence of the human TLR8 gene linked to the luciferase reporter [27]. The TLR8 agonist CL075 significantly increased the luciferase reporter activity in the monocytes transfected with the TLR8-luciferase reporter plasmid (**Figure 6C**). Treatment with 1,25(OH)_2_D_3_ significantly reduced the agonist-induced TLR8 promoter activation (**Figure 6C**).

### 1,25(OH)_2_D_3_ Inhibited Expression of TLR8 Downstream Mediators in THP-1 Monocytes Challenged with TLR8 Ligands

**Figure 7:**
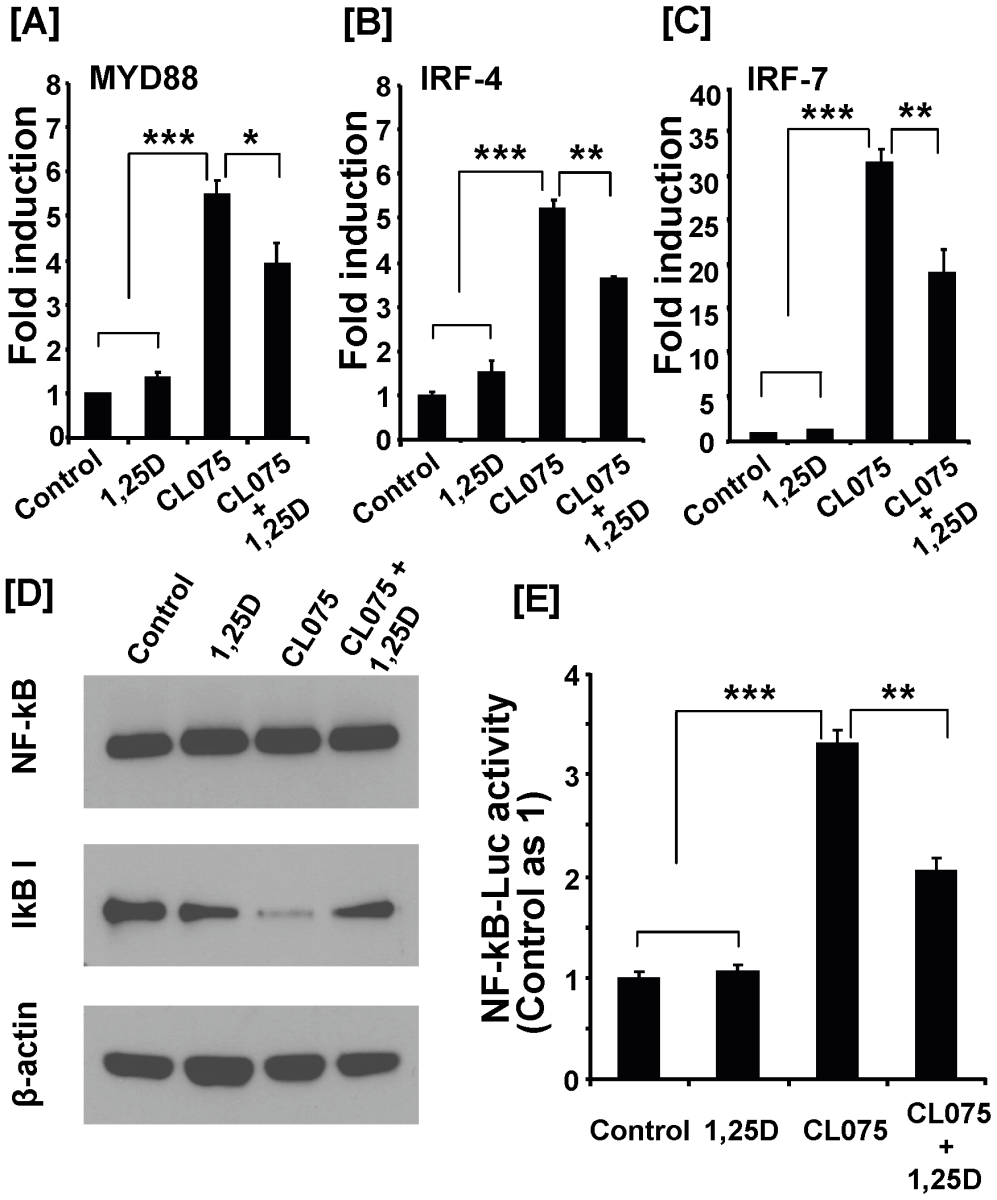
1,25(OH)_2_D_3_ inhibits TLR8 agonist-induced expression of key mediators of TLR8 signaling in human monocytes. (**A–C**) THP-1 cell treatment was described in Figure 5A–B legend. Cells were collected and subjected to RNA and protein extraction. mRNA levels of MyD88 (**A**), IRF-4 (**B**) and IRF-7 (**C**) were determined by real-time RT-PCR. Data represent means ± SEM (n = 4) and are representative of three independent experiments. (**D**) Western blotting for NF-kB and IkB-I protein detection was performed on total cell lysates from THP-1cells stimulated with CL075 (1 µg/ml) in the presence of vehicle or 1,25(OH)_2_D_3_ (100 nM) for 20 h. Representative results were obtained from two independent experiments. (**E**) The THP-1 cells were co-transfected with NF-kB promoter luciferase and TK-Renilla plasmid (*Materials and Methods*). After 24 h, the cells were pretreated with 1,25(OH)_2_D_3_ (100 nM) for 30 min and then stimulated with CL075 (4 µg/ml) for 20 h. Luciferase activity was measured and presented as the ratio of luciferase activity to TK-Renilla activity. The data shown are means ± SEM (n = 4) and are representative of three independent experiments. * *P*<0.05; ** *P*<0.01; *** *P*<0.001.

To further determine the mechanism underlying the inhibitory action of 1,25(OH)_2_D_3_ on inflammatory response in monocytes, we evaluated whether 1,25(OH)_2_D_3_ modulates expression or activity of key TLR8 downstream mediators in addition to TLR8 itself. 1,25(OH)_2_D_3_ treatment significantly reduced expression of MyD88 and its interacting proteins IRF-4 and IRF-7 (**Figure 7A-C**). Since TLR8 signaling requires activation of NF-kB pathway, we also evaluated whether components of this pathway were modulated by 1,25(OH)_2_D_3_. The total cellular level of NF-kB, evaluated by Western immunoblot analysis, was not affected by various treatments (**Figure 7D**). The cellular level of IkB, an inhibitor of NF-kB, was reduced by TLR8 agonist treatment (**Figure 7D**). Co-treatment with 1,25(OH)_2_D_3_ partially reversed the decrease in IkB level resulting from TLR8 activation(**Figure 7D**). Consistently, treatment with 1,25(OH)_2_D_3_ inhibited NF-kB activation as NF-kB-mediated luciferase reporter activity was significantly inhibited by 1,25(OH)_2_D_3_ treatment (**Figure 7E**). These data suggest that 1,25(OH)_2_D_3_ not only suppresses expression of TLR8 itself but also the expression or activity of its downstream mediators either directly or indirectly, leading to inhibition of inflammatory cytokine production.

## Discussion

Preclinical studies have shown that 1,25(OH)_2_D_3_ treatment effectively ameliorated EAE in mice [6], [7], [8], [9], [10], [11]. However, the mechanism by which this hormone provides the therapeutic effects has not been fully understood. Our findings in this study from both in vivo and in vitro experiments suggest that TLR8 is potentially a new target of 1,25(OH)_2_D_3_ and may, in part, mediate the anti-inflammatory action of 1,25(OH)_2_D_3_.

The murine TLR8 was once suggested to be biologically inactive based on the finding that viral ssRNA, the natural ligand for human TLR7/8, failed to activate mouse TLR8 [31]. The lack of mouse TLR8 activation by human TLR7/8 agonist was postulated to be due to the structural difference in the ligand binding domain of TLR8 between these two species [32], [33]. This prediction was consistent with the finding that a combination of TLR7/8 agonist and polyT oligodeoxynucleotides, was able to enhance activation of NF-kB in the human embryonic kidney 293T (HEK293T) cells overexpressing the mouse TLR8 [32], [33]. Now there is compelling evidence, which indicates that mouse TLR8 is indeed expressed and biologically functional in mouse tissues, especially in the central nerve system [22], [24]. Thus, a mouse EAE model is useful in evaluating the role and regulation of TLR8 in EAE.

To our knowledge, this is the first study which demonstrates that 1,25(OH)_2_D_3_ treatment effectively suppressed TLR8 expression in an autoimmune condition. This suppressive effect is likely to be mediated by both of the following mechanisms. First, the decrease in TLR8 mRNA level in the spinal cord is at least in part due to the decrease in the number of spinal cord cells expressing TLR8 (**Figure 3C–D**). This conclusion is consistent with the data showing that 1,25(OH)_2_D_3_ treatment remarkably reduced the abundance of infiltrating macrophages (**Figure 2A**) which are known to abundantly express TLR8 [22], [23]. Second, 1,25(OH)_2_D_3_ may directly act on the infiltrating inflammatory cells and/or resident microglia cells to inhibit their TLR8 expression and our in vitro data support this contention. The homologous up-regulation of the TLR8 expression by their synthetic or natural ligand was strongly inhibited by 1,25(OH)_2_D_3_ (**Figure 6A–B**). This inhibition could occur as early as 12 hours post-1,25(OH)_2_D_3_ treatment (data not shown). The 1,25(OH)_2_D_3_ inhibition of the TLR8 expression in monocytes is biologically significant as it links to an inhibition on both NF-kB and IRF-7 pathways and reduction in the expression of inflammatory cytokines TNF-α and IL-1β. Hence, our data suggest that 1,25(OH)_2_D_3_ suppression of TLR8 activation in EAE mice may have resulted from a direct inhibition on TLR8 signaling pathway within the infiltrating monocytes and/or activated microglia cells in the spinal cord lesion.

To begin to understand the mechanism by which 1,25(OH)_2_D_3_ suppresses the ligand-induced TLR8 expression in monocytes, we evaluated whether this regulation could occur at the transcriptional level. Consistent with a recent report [27], treatment of human monocytes with TLR8 agonist significantly increased the transcriptional activity of the 3.8 kb distal human TLR8 promoter. The agonist-induced TLR8 promoter activation is significantly inhibited by 1,25(OH)_2_D_3_ treatment. These data provide evidence that 1,25(OH)_2_D_3_ can acutely regulate TLR8 expression at the transcriptional level and support our premise that 1,25(OH)_2_D_3_ down-regulation of the TLR8 expression in the EAE spinal cord is in part mediated through a direct action of 1,25(OH)_2_D_3_ on inflammatory cells. 1,25(OH)_2_D_3_ exerts its biological functions through vitamin D receptor (VDR). Analysis of the 3.8 kb proximal TLR8 promoter sequence did not reveal presence of a consensus VDR response element. Thus, future studies are needed to determine whether VDR could interact with transcription factors regulating TLR8 transcription, such as C/EBPδ [27], leading to a decrease in TLR8 gene transcription.

In summary, this study provides strong evidence that 1,25(OH)_2_D_3_ regulates TLR8 expression and signaling in EAE leading to inhibition of the TLR8-mediated inflammatory responses. While our novel findings suggest that TLR8 activation may play an important role in initiation/propagation of EAE and potentially mediate the anti-inflammatory action of 1,25(OH)_2_D_3_ in CNS, this premise needs to be confirmed in future by more definitive studies. Such studies may include use of TLR8 knockout mice to determine if susceptibility to EAE could be reduced in the absence of TLR8 in knockout mice. Also, the TLR8 knockout mouse model could be employed to define if TLR8 plays an critical role in 1,25(OH)_2_D_3_ suppression of EAE. If this is the case, one would expect 1,25(OH)_2_D_3_ to be ineffective to ameliorate EAE in TLR8 knockout mice. Then therapy aimed at reducing TLR8 expression or activation could be developed to treat inflammatory neurological diseases including MS. Regarding the systemic 1,25(OH)_2_D_3_ therapy of multiple sclerosis, it is currently avoided to use in patients because the doses needed to achieve therapeutic efficacy is accompanied with hypercalcemia, which is an unacceptable side effect [34]. Future clinical application of this therapy will rely on development of 1,25(OH)_2_D_3_ analogues which retain immunosuppressing activity but will not cause hypercalcemia. More practically, targeting this therapy specifically to diseased organs may ameliorate the disease without causing systemic hypercalcemia.

## Supporting Information

Figure S1 **The disease courses in both prevention and treatment models.** Mice immunization and treatments were described in *Material & Methods*. Clinical EAE scores were shown at various time points after immunization with MOG in prevention model **(A)** and treatment model **(B)**. Data shown are means ± SEM (n = 4-5). * *P*<0.05; ** *P*<0.01.
(TIFF)

Figure S2 **1,25(OH)_2_D_3_ treatment ameliorated EAE and reduced expression of the MyD88 and IRF-7 in the treatment model.** Mice were injected with 200 ng 1,25(OH)_2_D_3_ every 3 days beginning at day 7 post MOG immunization. Experiment was terminated at day 14 post-MOG injection. **(A)** Average clinical EAE scores. **(B)** Real-time RT-PCR analysis of IL-17 mRNA level in spinal cords. **(C)** Concentration analysis of inflammatory cytokine IL-17 in the serum by ELISA. **(D-E)** Real-time RT-PCR analysis of MyD88 **(D)** and IRF-7 **(E)** mRNA levels in spinal cords. Data shown here (means ± SEM, n = 4−5) are representative of two independent experiments. * *P*<0.05; ** *P*<0.01; *** *P*<0.001.
(TIFF)

Figure S3 **Specificity of human TLR8 agonist on TLRs in human THP-1 monocytes.** Human THP-1 monocytes were pretreated with 1,25(OH)_2_D_3_ (100 nM) for 30 min and then stimulated with CL075 (1 µg/ml) for 20 h. mRNA levels of TLR4, TLR7 and TLR8 were subsequently determined by real-time RT-PCR. Data represent the means ± SEM (n = 4) and are representative of three independent experiments. ** *P*<0.01; *** *P*<0.001.
(TIFF)

Table S1 Primers used in this study for real-time RT-PCR.
(DOC)
