# Supplementary figures and images for: 1,25-Dihydroxyvitamin D3 Suppresses TLR8 Expression and TLR8-Mediated Inflammatory Responses in Monocytes In Vitro and Experimental Autoimmune Encephalomyelitis In Vivo
Source: PLoS One. 2013 Mar 14;8(3):e58808. doi: 10.1371/journal.pone.0058808 (PMC3597563; doi:10.1371/journal.pone.0058808)

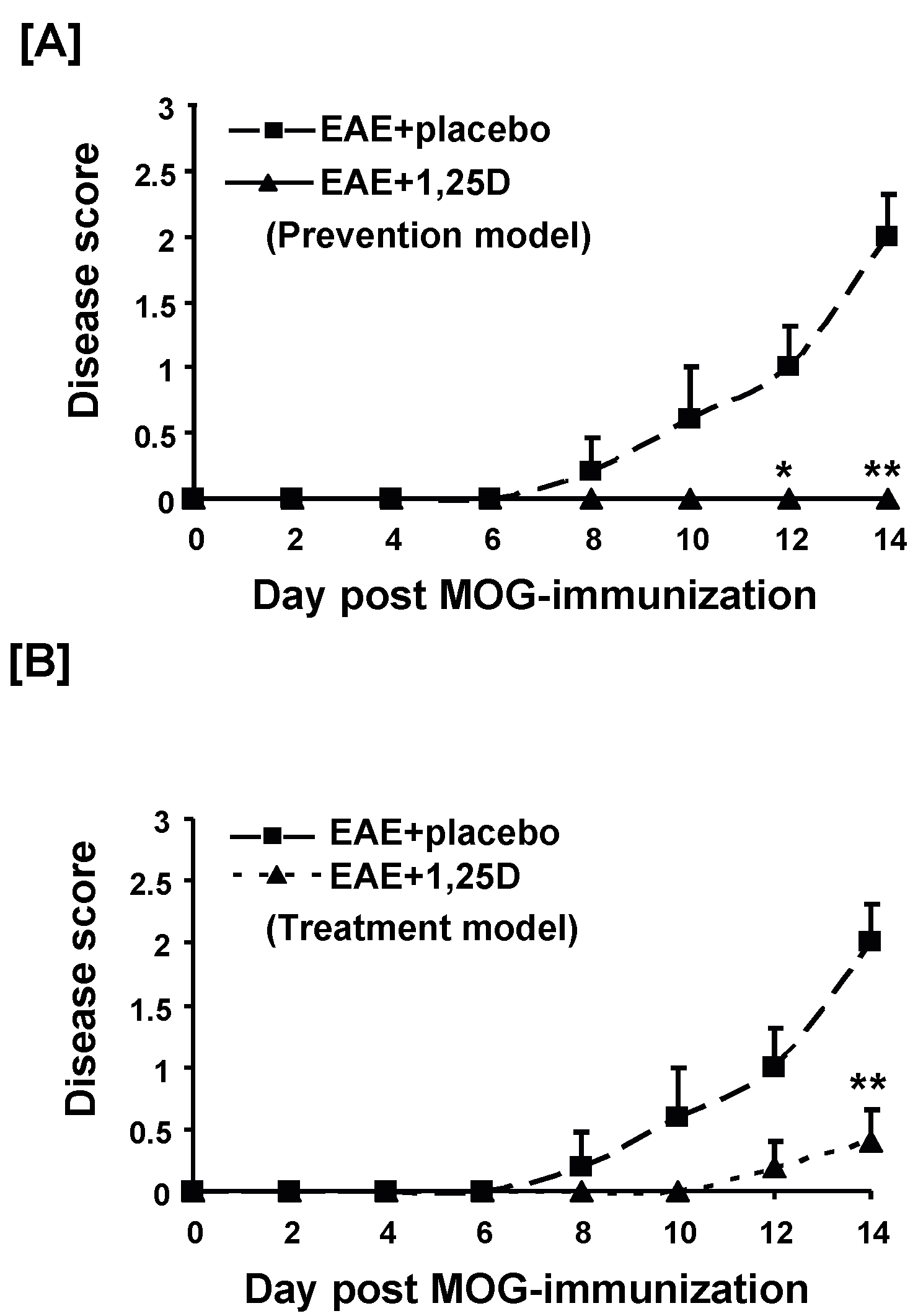

Supplement: Figure S1 — The disease courses in both prevention and treatment models. Mice immunization and treatments were described in Material & Methods . Clinical EAE scores were shown at various time points after immunization with MOG in prevention model (A) and treatment model (B). Data shown are means ± SEM (n = 4-5). * P<0.05; ** P<0.01. (TIFF) [file pone.0058808.s001.tiff]

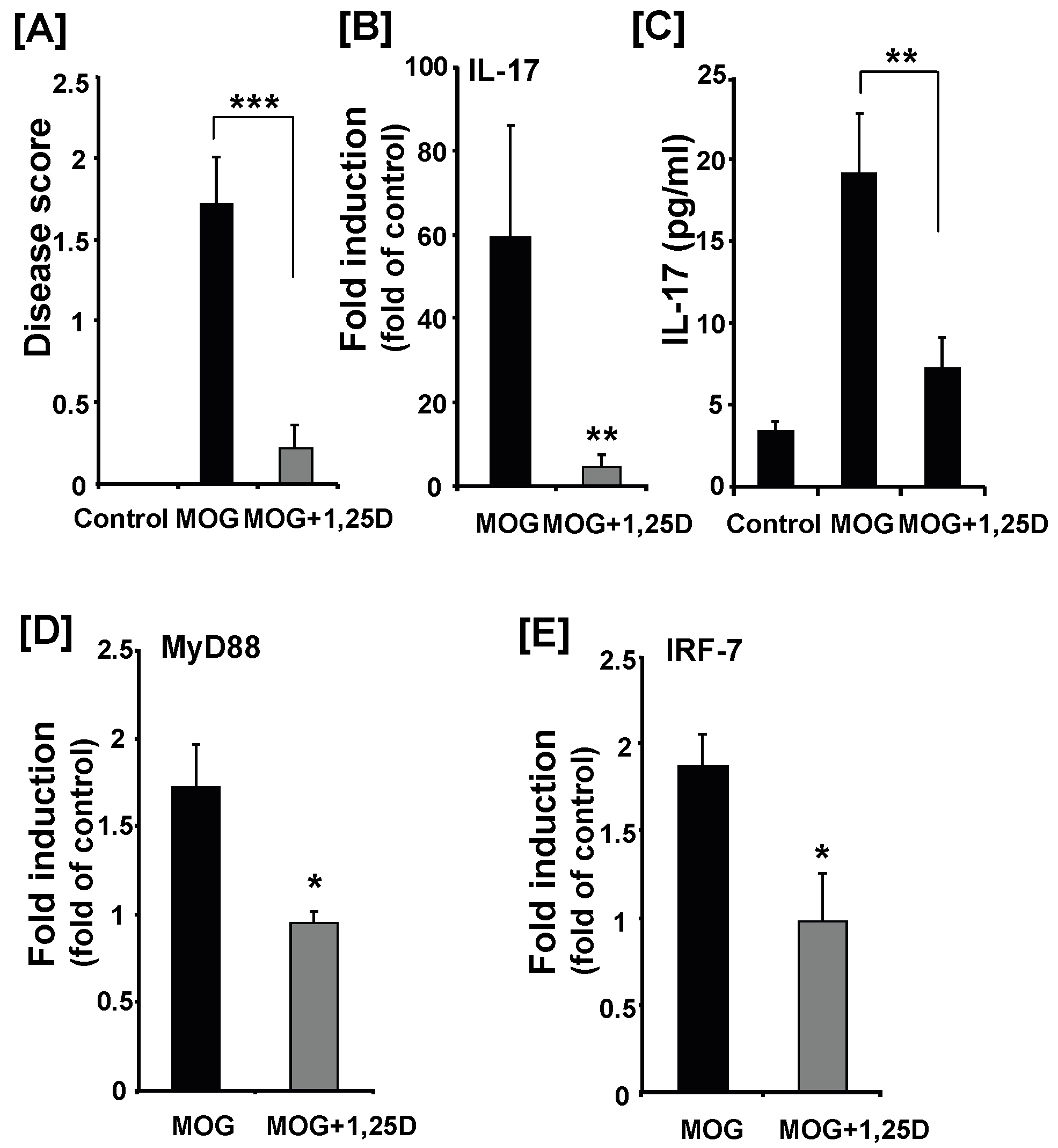

Supplement: Figure S2 — 1,25(OH)2D3 treatment ameliorated EAE and reduced expression of the MyD88 and IRF-7 in the treatment model. Mice were injected with 200 ng 1,25(OH)2D3 every 3 days beginning at day 7 post MOG immunization. Experiment was terminated at day 14 post-MOG injection. (A) Average clinical EAE scores. (B) Real-time RT-PCR analysis of IL-17 mRNA level in spinal cords. (C) Concentration analysis of inflammatory cytokine IL-17 in the serum by ELISA. (D-E) Real-time RT-PCR analysis of MyD88 (D) and IRF-7 (E) mRNA levels in spinal cords. Data shown here (means ± SEM, n = 4−5) are representative of two independent experiments. * P<0.05; ** P<0.01; *** P<0.001. (TIFF) [file pone.0058808.s002.tiff]

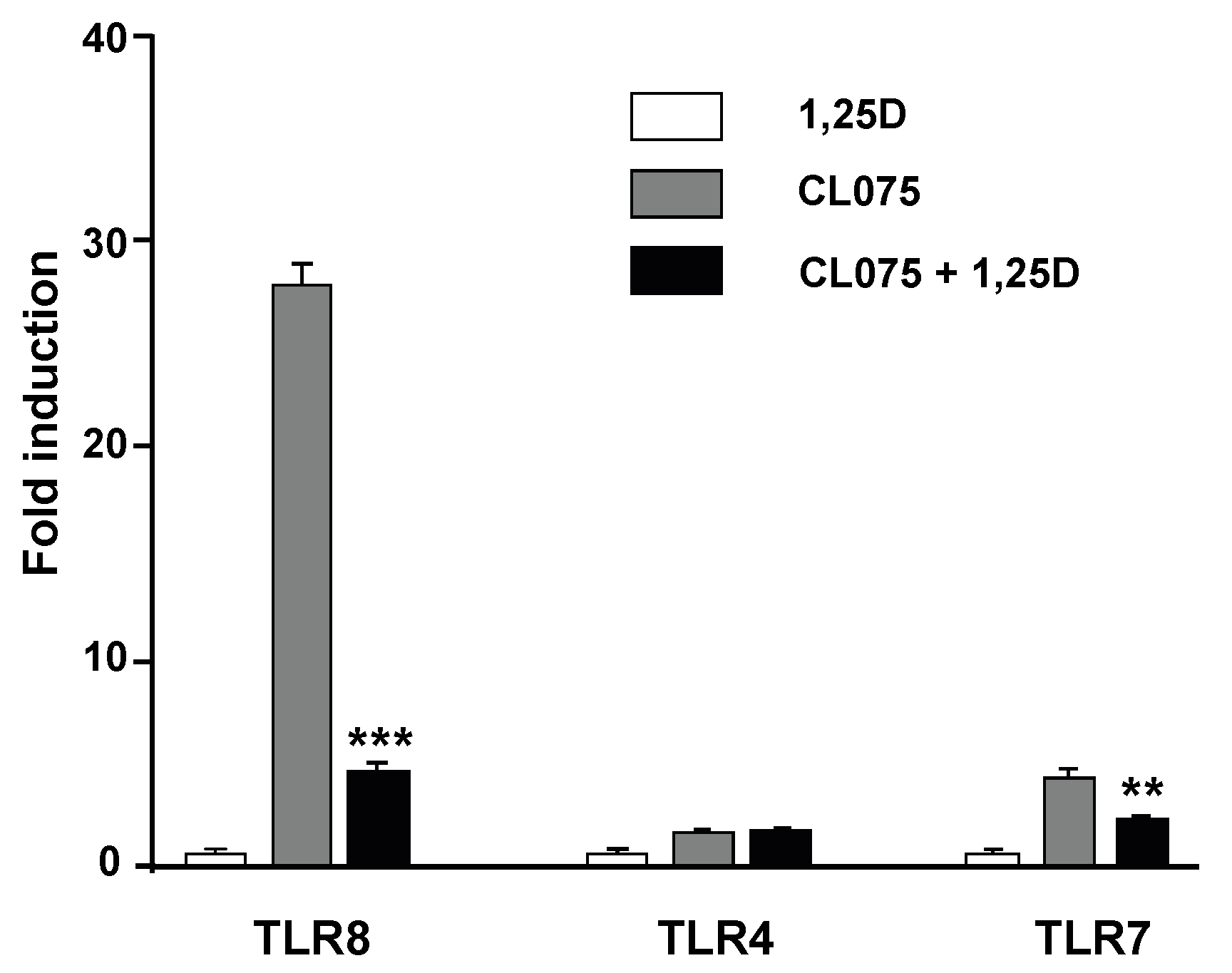

Supplement: Figure S3 — Specificity of human TLR8 agonist on TLRs in human THP-1 monocytes. Human THP-1 monocytes were pretreated with 1,25(OH)2D3 (100 nM) for 30 min and then stimulated with CL075 (1 µg/ml) for 20 h. mRNA levels of TLR4, TLR7 and TLR8 were subsequently determined by real-time RT-PCR. Data represent the means ± SEM (n = 4) and are representative of three independent experiments. ** P<0.01; *** P<0.001. (TIFF) [file pone.0058808.s003.tiff]
