# Supplementary material for: 1,25-Dihydroxyvitamin D3 Suppresses TLR8 Expression and TLR8-Mediated Inflammatory Responses in Monocytes In Vitro and Experimental Autoimmune Encephalomyelitis In Vivo
Source: PLoS One. 2013 Mar 14;8(3):e58808. doi: 10.1371/journal.pone.0058808 (PMC3597563; doi:10.1371/journal.pone.0058808)
Supplement: Table S1 — Primers used in this study for real-time RT-PCR. (DOC) [file pone.0058808.s004.doc]

| **Genes Forward Reverse** | | |
| --- | --- | --- |
| For mouse genes: | | |
| *TNF-α* | GAACTCCAGGCGGTGCCTAT | TCGGCTGGCACCACTAGTTG |
| *INFγ* | GATGCATTCATGAGTATTGCCAAGT | GTGGACCACTCGGATGAGCTC |
| *IL-17* | CTCCAGAAGGCCCTCAGACTAC | GGGTCTTCATTGCGGTGG |
| *TLR7* | GGGCATTCCCACTAACAC | GTTAGCCTCAAGGCTCAG |
| *TLR8* | AGTTGGATGTTAAGAGAGAAACAA | ATGGCACTGGTTCCAGAGGA |
| *TLR4* | TTGACACCCTCCATAGAC | CACCAAGAACTGCTTCTG |
| *TLR3* | TGTCTTCTGCACGAACCTG | CGCAACGCAAGGATTTTATT |
| *MyD88* | AAGAAAGTGAGTCTCCCCTC | TCCCATGAAACCTCTAACAC |
| *IRF-4* | AATGGGAAACTCCGACAGTG | TAGGAGGATCTGGCTTGTCG |
| *IRF-7* | GGTGGTTATCTGCAGTAGA | ATCTGTGTCCAGGGATAG |
| *IBP* | GGTGTCCAGTCAAGGTTA | GGAGCTTCTTCAGCAGAT |
| *Schlafen-4* | GCCCTCTGTTCAAGTCAAGTGTCC | CCCAGATGAAATCCTTTCCACGA |
| *GAPDH* | TGGCAAAGTGGAGATTGTTGCC | AAGATGGTGATGGGCTTCCCG |
| For human genes: | | |
| *TNF-a* | AGGCGGTGCTTGTTCCTCAG | CTCTCAGCTCCACGCCATTG |
| *IL-1**β* | AAGGCGGCCAGGATATAACT | CCCTAGGGATTGAGTCCACA |
| *TLR7* | TCTACCTGGGCCAAAACTGTT | GGCACATGCTGAAGAGAGTTA |
| *TLR8* | AACTTTCTATGATGCTTACATTTCTTATGAC | GGTGGTAGCGCAGCTCATTT |
| *TLR4* | CAGAGTTGCTTTCAATGGCATC | AGACTGTAATCAAGAACCTGGAGG |
| *MyD88* | CGGATGGTGGTGGTTGTCTC | CGCTTCTGATGGGCACCT |
| *IRF-4* | GAAGCCTTGGCGTTCTCAGAC | CGTATGTCCATGGGAGATCCG |
| *IRF-7* | TGGTCCTGGTGAAGCTGGAA | GATGTCGTCATAGAGGCTGTTGG |
| *β-actin* | TGAAGTGTGACGTGGACATC | ACTCGTCATACTCCTGCTTG |
